# Determinants of husbands’ involvement in family planning: Evidence from a community-based cross-sectional study in Uttar Pradesh, India

**DOI:** 10.1371/journal.pone.0343591

**Published:** 2026-04-29

**Authors:** Sanjiv Singh, Arupendra Mozumdar, Abhishek Kumar, Abhishek Gautam, Kaushalendra Kumar Singh, Rajib Acharya

**Affiliations:** 1 School of Business, University of Petroleum and Energy Studies, Dehradun, India; 2 Connexi, New Delhi, India; 3 PopulationCouncil Consulting Pvt. Ltd., New Delhi, India; 4 International Center for Research on Women, New Delhi, India; 5 Department of Statistics, Banaras Hindu University, Varanasi, India; 6 Population Council, New Delhi, India; Indian Institute of Dalit Studies (IIDS), INDIA

## Abstract

We argue that the husband’s involvement in family planning (FP) should encompass a larger role such as participation in the joint decision-making on FP and supporting their wives in contraceptive use, rather than just being FP clients. This study assessed husbands’ involvement in decision-making of family size and contraceptive use and examined factors associated with such involvement in two districts of Uttar Pradesh, India among husbands of 1,258 currently married women of reproductive age. The study considered a set of demographic, socio-economic, attitudinal, and programmatic variables to explain the variation in the husbands’ involvement in FP. We have applied latent class analysis to identify classes based on the attitude of husbands towards FP and contraceptive use. Nearly two-thirds (65%) of the husbands are involved in decision-making on family size and over half (56%) of the husbands are involved in supporting their wives’ contraceptive use. Having a positive attitude toward FP and contraceptive use along with media exposure to FP were the most significant determinants for husbands’ involvement in FP to decide family size (AOR = 4.18, 95% CI 2.96–5.88) and support wives’ contraceptive use (AOR = 3.98, 95% CI 2.82–5.61) even after adjusting for the factors like parity, religion, and having a son. The FP program should consider strategies involving husbands with positive attitudes across social and religious groups to popularize FP.

## 1. Background

Reproduction is increasingly recognized as a shared responsibility among couples rather than the just women’s responsibility. This principle got support in international platforms such as the International Conference on Population Development, held in Cairo, in 1994 and World Conference on Women in Beijing in 1995. The Government of India, as a key participant in both the conferences, recognizes several pivotal roles for men to be involved in FP programs—as initiators, as supportive partners, as the main responsible persons for the welfare of their families, and as torchbearers of transformation [1].

“Male involvement in family planning (FP) means more than just increasing the number of men using condoms or having vasectomies; male involvement also includes the number of men who encourage and support their partners to use FP and who influence the policy environment to be more conducive for developing male-related programs” [2]. Despite the policy shift at global and national level, FP programs in developing countries primarily focuses on women due to the biological risks of pregnancy and childbirth. However, the FP programs of the developing countries often kept out the men because program manager perceive that power inequalities between men and women in decision making on reproductive health has significant constraints to women’s access to FP services. In patriarchal settings, men frequently retain decision-making authority over reproductive choices, including the timing of intercourse and contraceptive adoption [3–5]. Consequently, by excluding men from counseling and information channels, programs inadvertently reinforce barriers to access, as men may perceive reproductive health services as insensitive to their needs or feel alienated from the process [6].

Based on these contrasting observations, the attempt to involve men in FP programs is based on a conceptual framework, namely ‘Women’s Rights and Men’s Responsibilities’, described by Basu [7]. This framework addresses the rights and responsibilities differently for women and men, acknowledging the difference in power dynamics between them. The literature suggests that the most successful FP programs target couples as an unit to counsel them about on reproductive health, fertility, availability of contraceptive method, and its adoption [8–9]. The literature also demonstrated that effective involvement of husband in reproductive health and FP have positive health outcomes for both the mother and child [10].

In spite of the promising outcomes, the desired level of male involvement is yet to be achieved. The recent evidence shows that use of male methods of modern contraception in India remains miniscule-only 9.5% of Indian women reported that their husbands/partners use condoms and merely 0.3% have undergone male sterilization (vasectomy), despite its simpler and less risky procedure than that of female sterilization (tubectomy) [11,12].

Historically, efforts to involve men in FP have focused to consider them as clients or users of contraception [13]. While the literature is replete with studies determining factors of male contraceptive use [14–19], there is a distinct scarcity of research examining men’s involvement as supportive partners who facilitate their wives’ contraceptive choices. In social settings of historic trend of low usage of male methods, yet having higher fertility, it is programmatically challenging to convert men into users, rather it is much easier for the program to engage men as a partner and encourage them to play the role of the partner, responsible for the wellbeing of their wives and their families [20–22].

Earlier studies recognized effective male involvement must extend beyond method uptake to include the encouragement and support of female partners [23–24]. This distinction is critical in high-fertility regions like Uttar Pradesh, India where male attitudes often dictate women’s ability to act on their reproductive desires [25]. Men’s attitude toward FP and contraceptive use therefore become a crucial factor that may be dominating over women’s empowerment regarding FP choices [23,26], the literature review demonstrated that very few research attempted to study men’s involvement as equal and supportive partners of contraceptive use of their wives and its determinants. Addressing this research gap, this study moves beyond the traditional focus on male method use. Instead, it investigates the factors associated with husbands’ behavior as an equal and supportive partner in FP in select districts of Uttar Pradesh.

## 2. Method

### 2.1 Conceptual framework

Basu’s [7] “Women’s Rights and Men’s Responsibilities” framework criticizes existing FP programs which keep men away by segregating roles and failing to involve men actively in interventions; these programs miss the opportunity to transform the underlying social dynamics necessary for effective family planning. The Theory of Planned Behavior (TPB), a widely applied behavioral theory, provides the mechanism to address this gap by focusing on the determinants of an individual’s intention to perform a specific behavior (e.g., supporting decision-making and contraceptive use). Instead of viewing male involvement purely through the lens of Gender and Power Theory, which focuses on structural inequalities, TPB allows us to analyze how those power dynamics and gendered expectations translate into the psychological determinants of an individual man’s intention to engage in family planning, shifting the focus from structural inequality to the individual psychological impact of that inequality. The TPB framework also allows us to explore how gender roles shape attitudes and perceived control. By focusing on these determinants, we aim to provide evidence for designing inclusive interventions that successfully shift subjective norms toward shared responsibility, thereby fulfilling the potential for greater success that Basu advocates for [7].

**Fig 1:**
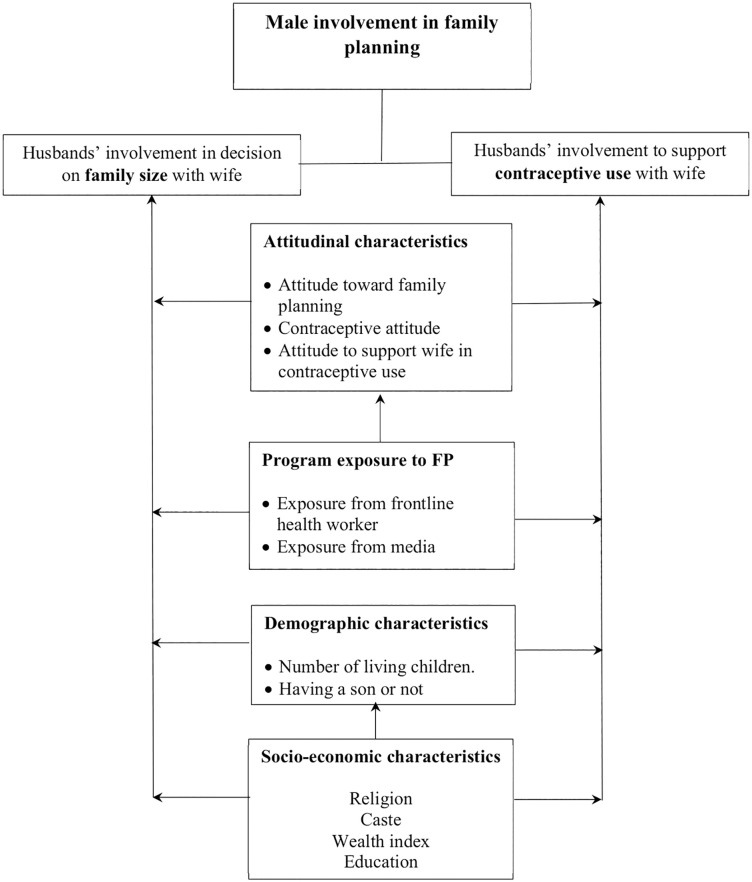
Conceptual framework for male involvement in family planning.

Based on the findings of the earlier studies, we conceptualized a framework that explains the male involvement in FP as a responsible partner and its determinants (Fig 1). We studied two types of men’s behaviors related to their involvement in FP: (1) husbands’ involvement in making decisions on family size with their wives, and (2) husbands’ involvement in supporting their wives in contraceptive use. Importance of these behaviors instead of the mere contraceptive use among men has been proposed by earlier research [3,6,27,28].

In our framework, a ‘husband-only’ decision is conceptualized as an indicator of a male-dominated, non-equitable power dynamic, which is a known barrier to women’s reproductive health and autonomy [29,30]. Therefore, the outcome variable husbands’ involvement in making decisions on family size with their wives denotes the extent to which the husband participates in or supports an equitable, non-coercive decision-making process regarding the number of children, respecting the wife’s autonomy. We provided specific items used for measurement, and the precise coding rules for this variable in S1 Appendix.

The other outcome variable, husbands’ involvement in supporting their wives in contraceptive use, indicates husband’s attitudinal and practical support for his wife’s use of family planning methods to prevent or delay pregnancy. The specific items and coding rules of this variable are provided in Appndix-1. We considered a ‘supportive’ male involvement for the cases of ‘joint decisions’ as supportive as this represents the ideal of an equitable, communicative partnership often promoted in family planning programs. We also coded ‘wife-only decisions’ as ‘supportive male involvement’ because this operationalization defines ‘support’ not just as active participation, but also as respect for the wife’s autonomy. In this scenario, the husband is ‘involved’ by supporting his wife’s agency to make decisions about her own reproductive life. This aligns with a human rights-based approach to family planning, which posits that a woman has the right to make these decisions, and a supportive partner is one who respects and enables that right, even if he is not the primary decision-maker [31].

By defining our variables in this way, we establish two distinct outcomes. The first variable, husbands’ involvement on decision making of household-size, measures the locus of power in a long-term reproductive goal. While the other variable, husbands’ involvement in supporting their wives in contraceptive use measures the husband’s active attitudinal approval of the specific behavior (contraceptive use) required to meet that goal. This distinction allows us to separately analyze how a husband’s characteristics might influence the general decision-making environment versus his specific approval of contraceptive use as a means to achieve their reproductive goal.

To guide the analysis of male involvement in family planning, our conceptual framework anchored in the Theory of Planned Behavior (TPB), a widely applied behavioral theory. In the context of family planning, TPB provides a robust lens to understand husbands’ involvement in decision-making about family size and support for contraceptive use. By grounding the framework in TPB, we perceive that the coherent pathways to male involvement will begin with their intention to use contraceptive. In Indian context, demographic characteristics of number of living children, especially number of sons determine couples’ use and intention to FP [32,33]. These demographic characteristics often strongly associated to the socio-economic characteristics like religion, caste, wealth index, and education [34–37].

People with similar demographic and socio-economic characteristics can behave differently based on their attitude and their mental make-up [26,28,38]. We perceive the husband’s attitude toward FP, contraceptive use, and the attitude to support his wife in her contraceptive use would determine his involvement in FP. Exposure to FP messages, delivered by health professionals or through various media channels, acts as behavioral control and can improve both attitudes towards and perceived benefits of FP among men [39–41]. Therefore, in the conceptual framework we perceived that outcome variables that indicate male involvement will be affected by attitude of husbands which will be shaped by their exposure to FP program through outreach and media.

### 2.2 Study settings

This cross-sectional study aimed to quantitatively measure husbands’ involvement in FP and identify its key determinants in two districts—Aligarh and Fatehpur—of Uttar Pradesh, the most populous state in India. Uttar Pradesh had a population of 199.8 million, total fertility rate of 2.4, and modern contraceptive prevalence rate (mCPR) of 44.5% with total unmet need of 12.9%.[12–42] Aligarh and Fatehpur have a population of 3.7 million and 2.6 million, and mCPR of 39.7% and 58.0% respectively. FP use among men was low in both the districts, with male sterilization rates of 0.3% in Aligarh and less than 0.01% in Fatehpur, and the condom use of 20.3% in Aligarh and 38.9% in Fatehpur [12].

### 2.3 Sampling strategy

This study surveyed married couples in Aligarh and Fatehpur districts of Uttar Pradesh, India during 2021. Primary participants of the study were wives within their reproductive years (15−49 years old), which were part of project involving longitudinal data collection with women. The sample size was determined considering program priorities, particularly focusing on young couples. Additionally, Aligarh, one of the study districts with a 49% urban population, had its sample size calculated separately for rural and urban areas. To estimate the response distribution, we utilized the proportion of women intending to use contraception in the next 12 months as the key indicator, disaggregated by age groups (15−24 years and 25−49 years), and residence (urban and rural), using the NFHS 2015−16 estimates. We calculated the final sample size based on the following assumptions—5% level of confidence, 80% power, 15% attrition considering lost to follow-up in the subsequent round, 1.5 design effect, and 15% non-response rate; which yield sample size of 1,100 women in Fatehpur and 1,200 in Aligarh. We planned to interview husbands of 50% of the women participants, therefore, a targeted sample for husbands were 550 in Fatehpur and 600 in Aligarh.

The estimated sample size was interviewed from primary sampling units (PSUs), which were villages in rural areas and census enumeration blocks (CEBs) in urban areas. After checking the distribution of married women of 15–24 years and 25–49 years, the researchers decided to sample 50 women from each PSU, ensuring each PSU had at least 250 households to provide the required 25 married women of younger age-group.

**Fig 2:**
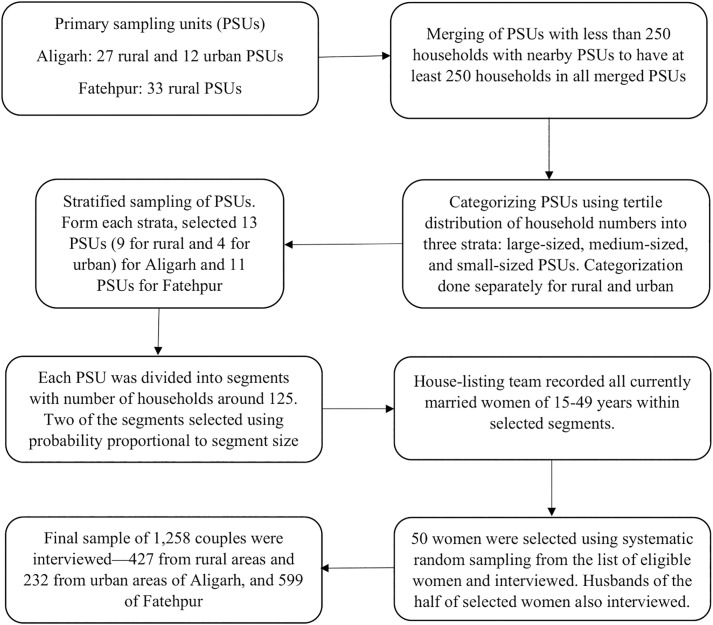
Flow diagram showing sampling and data collection process. * Primary Sampling Units (PSUs) were villages in rural areas and census enumeration blocks (CEBs) in urban areas.

We used the district census population figures to sample the PSUs. At first, all villages with less than 50 households were removed from the sampling frame and that excluded less than 2% of the total population. Then the PSUs with less than 250 households were merged with their neighboring PSU to create larger sampling units of at least 250 households. We then employed stratified sampling of PSUs using the tertile distribution of household numbers of PSUs, given in census, allowing equal number of large-sized, medium-sized, and small-sized PSUs, ensuring the representativeness of the sampled PSUs. The final sample design included 27 rural PSUs in Fatehpur and 33 in Aligarh, comprising 21 rural, and 12 urban sampling units. For ease of understanding, we presented a flow diagram of the sampling procedure (Fig 2).

### 2.4 Data collection

The PSUs were divided into segments with number of households around 125. Two of the segments were randomly selected from each PSU for the survey using systematic sampling with probability proportional to the segment size. Therefore, a selected sampling unit is either an entire PSU or a segment of a larger PSU. A house-listing team listed all households of the segment to identify a household with eligible women, i.e., currently married women of reproductive age (15–49 years). From the sampled households of PSU, using systematic random sampling, 50 women were selected for interviews. Of those 50 women whoever were available in the household at the time of visit and agreed to participate were interviewed by the female investigators. Among the women participants of the study, half of their husbands were also approached for participation, and those who agreed were interviewed. A total of 1,258 couples were interviewed: 427 from rural, and 232 from urban Aligarh and 599 from rural Fatehpur.

The survey utilized a computer assisted personal interviewing (CAPI) program, developed in CS-Pro software for data collection. To ensure participant comfort and cultural sensitivity, female investigators conducted interviews with wives, while male investigators interviewed their husbands. The research team trained field investigators in a weeklong program, which covered project background, objectives, data collection tools, personal interviewing technique, implementation of study tools using CAPI program installed in Android tablets, and practices for maintaining the data quality. The field team recruited participants from 13 February 2022 to 9 April 2022. Data on several aspects of family health, use of contraceptives, and attitude toward family planning has been collected by self-reporting, which has the possibility to be affected by social desirability bias, where respondents may indicate more positive behaviors or attitudes than they exhibit. To minimize such bias field investigators were intensively trained and care has been taken to maintain the privacy of the interviews in the field. Moreover, to ensure high-quality data collection, the research team implemented a multi-pronged approach. This included regular monitoring of collected data, holding of two feedback sessions during the course of the study: one in-person and the other online, gathering feedback from investigators, and consistent communication with field supervisors to oversee data collection processes.

### 2.5 Ethical consideration

The study was conducted in accordance with the ethical guidelines on human research of the Institutional Review Board (IRB), Population Council, New York. Before starting the fieldwork, the technical and program staff of the authors’ organizations internally reviewed the study proposal both for technical and ethical quality. A separate ethical approval was also received from the National Health Mission, Uttar Pradesh, subsequently the office of the chief medical officer (CMO) of the study districts approved the study. All participants were above 18 years of age, therefore, eligible to give consent for participation by themselves. Participants were informed about the purpose of the study before the interview. The consent form was in Hindi-the language of the study area-and each participant gave written consent before the interview. If a participant was illiterate, one of the community members who was literate witnessed the consent giving process, explained the information given in the consent form to the participants in the local language, and ensured that the participants understood their rights and voluntarily participated in the study.

### 2.6 Data processing

**Outcome variable**: This study considered two outcome variables—(1) husband’s involvement in decision on family size with wife, and (2) husband’s involvement to support wife in contraceptive use. The outcome variable on husband’s involvement in decision on family size with wife had been generated based on questions ‘Have you ever discussed number of children to have with your wife?’ and ‘Have you ever discussed about spacing children with your wife?’ of the men questionnaire. Husbands who answered ‘yes’ to both the questions had been considered involved in making reproductive preferences and coded as ‘1’, otherwise ‘0’. The outcome variable on husband’s involvement to support wife in contraceptive use was generated based on three questions regarding discussion on contraceptive use. The first question was asked whether the husband ever discussed contraceptive methods with wife. The second set of questions were asked to two sets of respondents-one who is currently using any contraceptives and the other one who is not using any contraceptives and not pregnant. Those who were using any contraceptive at the time of survey were asked “Would you say that using contraception was mainly your decision, mainly your wife’s decision, or you both decided together or someone else took the decision?”. Similarly, those who are not using any contraceptive were asked “Would you say that not using any contraception is mainly your decision, mainly your wife’s decision, or you both decided together or someone else took the decision?” Answer choices to both these questions were ‘responded’, ‘respondent’s wife’, ‘joint’, or ‘someone else’. Husbands who reported discussing contraceptive use with wives, where the main decision was made either by the wife or jointly, were considered as involved in supporting wife’s contraceptive use and coded as ‘1’, otherwise were coded as ‘0’.

**Independent variables**: As shown in Fig 1, this study considered four sets of explanatory variables: demographic characteristics, socio-economic characteristics, attitudinal characteristics, and program exposure to FP. The data processing procedures for generating each set of variables are described below.

***Demographic characteristics:*** This study considered two demographic characteristics as determinants of participants’ contraceptive behaviors: the number of living children and the number of sons. The variable for ‘number of living children’ was re-coded into following categories-‘no child’, ‘1 child’, ‘2 children’, and ‘3 or more children’. The variable for ‘number of sons’ was categorized into ‘no son’, ‘1 son’, and ‘2 or more sons’.

***Socio-economic characteristics***: The socio-economic characteristics that have been considered in the analyses were religion, social group, household wealth, and education status of the respondents. Religious affiliation of the participants was categorized as ‘Hindu’ or ‘Non-Hindu,’ which mostly included the Muslims. The respondents identified themselves as belonging to either Scheduled Caste or Scheduled Tribe (SC/ST), Other Backward Classes (OBC), or ‘general’. The household wealth index was computed using data on the household’s ownership of 33 select assets, and availability of household’s amenities and facilities. Households were given scores that were derived from adjusted weights using principal component analysis. Wealth terciles were compiled by assigning the household score to each respondent, and then dividing the distribution into three equal categories namely ‘poor’, ‘middle’, and ‘rich’, each with one-third of the population. Respondent’s education was recoded into four categories: No formal education, Primary (up to grade 5), Secondary (grade 6–10), and Higher (higher secondary certificate or above).

We examined the background characteristics (age groups, number of children, number of sons, religion, social group, and education status) of the women whose husbands participated in the study vs whose husbands did not. Except for the social group, the distribution of the background characteristics was not significantly different between the women of the two groups.

***Attitudinal characteristics:*** Three types of attitudinal characteristics of husbands have been considered: 1) husbands’ attitude towards FP, 2) husbands’ attitude towards contraceptive use, and 3) husbands’ attitude to support their wives in contraceptive use. Among these three types of attitudinal characteristics the husbands’ attitude towards contraceptive use was measured with a 13-item scale, on contraceptive attitude. This scale was developed by the research team adopting questions from standard scale or questionnaire used to measure attitudes towards contraceptive use, in various cultural contexts, particularly in studies concerning barriers to contraceptive use [43,44]. Although, the psychometric properties are not available, the scale was validated and culturally contextualized among a sample of 30 men living in Meerut district of Uttar Pradesh, India. Each item was a statement related to contraceptive use enquiring the attitude of the participants (husbands), who responded with ‘strongly agree’ ‘agree’, ‘disagree’, or ‘strongly disagree’. For ease of the analysis, we re-coded the responses into a binary scale, depending on the direction of the statement. For example, responses of ‘strongly agree’ or ‘agree’ to positive statements were coded as ‘1’, and ‘disagree’ and ‘strongly disagree’ were coded as ‘0’; negative statements were coded inversely. Following the re-coding, scores were summed up to get a total score for each respondent, ranging from 2 to 13, with a higher score indicating a more positive attitude and vice versa. Examining the distribution of the total score we classified respondents into two groups using “median split” method. We found that the 50^th^ percentile of the score belonged to scoring of 9, therefore, respondents who scored 2–8 were considered to have a ‘anti-contraceptive’ attitude, and those who scored between 9–13 were considered to have a ‘pro-contraceptive’ attitude towards contraceptive use.

Since no standard measure exist for the other two attitudinal variables—husbands’ attitude towards FP, and husbands’ attitude to support wives in contraceptive use, we used latent class analysis (LCA) to generate attitude scores and categorized husbands having either negative or positive attitude toward each construct [45]. The LCA is a measurement model that classifies individuals into mutually exclusive groups, called latent classes, based on their pattern of responses to a set of categorical indicator variables.

The assumption underlying LCA is that membership in unobserved groups (or classes) can be explained by patterns of scores across survey questions, assessment indicators, or scales. To detect the latent groups, LCA uses study participants’ responses to categorical observed variables [46]. It is often said that factor analysis is a “continuous form” of LCA or vice versa—both latent and unobserved variable is continuous in factor analysis and while those are categorical in LCA. Approach of factor analysis is “variable centered”, which uses correlation between two variables to calculate the continuous latent scores, whereas the approach of LCA is “person centered”, which groups individuals based on their response into categories. Latent profile analysis (LPA) is another similar statistical procedure that produces categorical latent classes, however, it deals with continuous observed variables whereas LCA deals with categorical observed variables [47].

LCA assumptions include the independence of observed variables given class membership and a finite mixture of distributions. LCA can identify groups with similar outcomes, leading to better-targeted interventions. The analysis accounts for the probabilistic nature of class membership, providing a more accurate representation of the population. The method also allows complex survey weighting to be applied during the modelling process to enhance the representativeness and accuracy of survey results.

A frequent issue in LCA is selecting the wrong number of latent classes, leading to overfitting or underfitting. To avoid this, use criteria like Akaike Information Criterion (AIC) and Bayesian Information Criterion (BIC) to find the optimal number of classes. Misinterpreting results is another disadvantage of LCA; however, researchers ensure the correct understanding and interpretation of the class memberships and their implications by examining the marginal plots showing the predicted probability of observed characteristics across latent groups.

The sets of questions to measure these two attitudinal characteristics of husbands are given in S1 Appendix. Two latent classes or groups of husbands were classified, namely ‘anti-FP’ and ‘pro-FP’ for attitude of husbands towards FP; and ‘non-supportive’ and ‘supportive’ for attitude of husbands to support their wives in contraceptive use.

***Exposure to messages on FP:*** We hypothesized that exposure to FP messages would increase the chance of husbands engaging in FP. Exposure to FP messages can occur through two channels: direct interactions with frontline health workers and various forms of media. Husbands who reported discussing FP either with an Accredited Social Health Activist (ASHA), Anganwadi Worker (AWW), or Auxiliary Nurse Midwife (ANM) in the last 12 months were considered as exposed to FP messages from frontline health workers (FLWs). Husbands who reported exposure to FP messages in the past 12 months through radio, television, newspapers, or any other visual media (paintings, hoardings) were considered exposed to media-based FP messages.

### 2.7 Statistical analysis

We conducted univariate analysis of both dependent and independent variables for the pooled sample and by district to profile the study participants. Bivariate analyses were conducted to examine the association between dependent variables and independent variables by estimating the distribution of the two dependent variables across various categories of independent variables. Multivariable logistic regression analyses were conducted for each of the two dependent variables considering all four types of independent variables-demographic, socio-economic, attitudinal, and exposure to FP messages-together in the model.

Two final models of multivariate logistic regression were applied to identify the factors determining husbands’ involvement in FP. These models considered variables with significant associations from previous regression models and included an interaction term between newly formed attitudinal factor and exposure to FP through media. This aimed to explore the combined effect of these two factors on husbands’ involvement in FP. We presented the results as adjusted odds ratios with 95% confidence intervals.

Stata 15.0 software was used for all the statistical analyses. To address missing values in the dataset, the pairwise deletion technique was employed. This method retains all available data for each analysis by excluding only those observations that have missing values for the specific variables involved in a given computation. We chose this approach for maximizing data utilization, preserving loss of sample size as much as possible, and ensuring that the analysis remained robust without discarding unnecessary information. The proportion of missing data was relatively low and appeared to be random.

## 3. Results

### 3.1 Characteristics of the study participants

Around 65% of husbands were involved in decision-making on family size with their wives, and approximately 56% supported their wives in contraceptive use (Table 1). While 60% of participants had two or more children, 68% had at least one son. Most participants (89%) were Hindus, 46% belonging to the OBCs, and 53% completed at least a secondary level of education. About 42% of husbands received FP information or messages through various media, only 8% reported receiving such messages from frontline health workers.

**Table 1:** Distribution of the study participants by their characteristics separated by districts.

	Fatehpur (%) (N = 599)	Aligarh (%) (N = 659)	Total (N = 1258)
**Dependent variables**			
Male involvement in decision on family size with wife	53.1	74.9	64.5
Male involvement to support contraceptive use with wife	46.8	65.3	56.3
**Explanatory variables**			
**Demographic characteristics**			
***Number of living Children***			
0	17.4	17.3	17.3
1	23.4	22.0	22.7
2	22.2	27.6	25.0
3+	37.1	33.1	34.9
**Number of sons**			
No son	33.1	31.7	32.4
One son	35.6	37.6	36.7
Two or More Son	31.4	30.7	31.0
**Socio-economic characteristics**			
***Religion***			
Hindu	89.8	88.8	89.3
Non-Hindu	10.2	11.2	10.7
***Social-group***			
Scheduled caste (SC)/ Scheduled tribe (ST)	31.4	27.8	29.5
Other backward classes (OBC)	41.2	50.2	45.9
General	27.4	22.0	24.6
***Wealth index***			
Poor	42.2	24.9	33.2
Middle	33.7	31.4	32.5
Rich	24.1	43.7	34.3
***Education status***			
No education	25.2	32.6	29.1
Primary	16.5	17.8	17.2
Secondary	33.9	30.9	32.4
Higher	24.4	18.7	21.4
**Attitudinal characteristics**			
***Attitude towards family planning***			
Anti-FP	34.9	32.9	33.9
Pro-FP	65.1	67.1	66.1
***Contraceptive Attitude***			
Anti-contraceptive	47.5	51.8	49.8
Pro-contraceptive	52.3	48.3	50.2
***Attitude to support wife in contraceptive use***			
Non-supportive	51.3	54.2	52.7
Supportive	48.8	45.9	47.3
**Exposure to family planning messages**			
***Exposure from frontline health worker***			
No exposure	92.7	91.5	92.1
Got Exposure	7.4	8.5	7.9
***Exposure from media***			
No exposure	62.4	54.5	58.3
Got Exposure	37.6	45.5	41.7

Note: Scheduled caste, scheduled tribes, and other backward classes are marginalized groups in India designated by the government and recognized by the Constitution of India

### 3.2 Creating latent class of the husbands using attitudinal characteristics

We initially created latent classes for two sets of attitudinal characteristics of husbands: attitude toward family planning and attitude to support wife in contraceptive use. We started to estimate LCA models to identify the optimal number of latent classes that best represents the distribution of husbands based on their attitudinal characteristics. However, for both sets of attitudinal variables the model beyond two classes did not converge. The results of the fit indices of LCA have been presented in S2 Appendix. The Akaike Information Criterion (AIC) and Bayesian Information Criterion (BIC) statistics showed considerable improvement in two class models from one class models. Since the models estimate two class models therefore, the 50^th^ percentile value for predicted probability has been considered as the cut-off to assign class membership of individuals. Further the marginal plots for mean posterior probabilities with 95% CIs have been created to present how the individual attitudinal characteristics are distributed by assigned latent class membership (S3 Appendix).

The distribution of predicted probabilities and the CIs for two classes of participants showed significant differences in their attitudinal characteristics. For the ease of understanding, we denoted the class of husbands’ showing lower mean values for predictive probabilities of the positive attitude toward FP as ‘anti-FP’ husbands and higher mean values as ‘pro-FP’. Among 1,258 husbands, the study participants, 34% of the husbands were classified having ‘anti-FP’ attitude while 66% as having ‘pro-FP’ attitude.

Similarly, we denoted the class of husbands showing lower mean values of predictive probabilities of the positive attitude to support wife in contraceptive use as ‘non-supportive’ while those showing higher mean value as ‘supportive’ husbands. The LCA classified 53% of husbands as ‘non-supportive’ and 47% as ‘supportive’ to their wives’ contraceptive use.

Unlike two above-mentioned attitudinal characteristics, the contraceptive attitude was measured using a scale on contraceptive attitude. Responses for each of the 13 items were recoded into 0 or 1. The sum of recoded scores ranged from 2 to 13 with a modal distribution at 8 and 9. We also found the median value, i.e., 50^th^ percentile of the score belong to scoring of 9, therefore, we classified the husbands who scored ‘0-8’ as having ‘anti-contraceptive’ attitude, while those who scored 9–13 as having ‘pro-contraceptive’ attitude. About half of the husbands were classified as having ‘pro-contraceptive’ attitude.

### 3.3 Bivariate association between husbands’ involvement in FP and explanatory variables

The participants with one child (75%) or two children (70%) are more involved in decision-making on family size with their wives (Table 2). Similarly, the participants with one son (72%) are more involved in decision-making on family size than the participants with no son or with two or more sons. Hindu participants from OBC backgrounds, living in well-off households with at least a higher secondary education, reported greater involvement in decision making about family size. Higher proportion of participants with ‘pro-FP’, ‘pro-contraceptives’, and ‘supportive’ attitude were involved in decision-making on family size compared to those with ‘anti-FP’, ‘anti-contraceptive’ and ‘non-supportive’ attitudes. Additionally, participants who had higher exposure to FP messages through FLWs or media were involved in decision-making in higher proportions than those with less exposure.

**Table 2:** Bivariate association of male involvement and explanatory variables.

	Dependent variables	
	Male involvement in decision on family size with wife (%)	Male involvement to support contraceptive use with wife (%)
**Explanatory variables**		
**Demographic characteristics**		
***Number of living Children***		
0	58.3	41.7
1	75.1	56.4
2	70.2	62.0
3+	56.8	57.3
**Number of sons**		
No son	64.1	48.4
One Son	72.0	60.9
Two or More Son	56.2	57.7
**Socio-economic characteristics**		
***Religion***		
Hindu	66.1	57.9
Non-Hindu	51.9	42.2
***Social-group***		
SC/ST	60.1	50.8
OBC	66.4	60.1
General	66.3	55.9
***Wealth index***		
Poor	55.6	53.8
Middle	64.3	53.9
Rich	73.4	61.3
***Education status***		
No education	55.2	49.1
Primary	60.7	56.7
Secondary	68.8	58.6
Higher	73.9	63.2
**Attitudinal characteristics**		
***Attitude towards family planning***		
Anti-FP	51.4	43.5
Pro-FP	71.3	62.9
***Contraceptive Attitude***		
Anti-contraceptive	56.3	48.6
Pro-contraceptive	72.2	64.0
***Attitude to support wife in contraceptive use***		
Non-supportive	61.1	45.3
Supportive	68.4	68.6
**Exposure to family planning messages**		
***Exposure from frontline health worker***		
No Exposure	63.6	55.1
Got Exposure	75.0	71.3
***Exposure from media***		
No Exposure	55.8	49.5
Got Exposure	76.8	66.4

The husbands with two children (62%) were more ‘supportive’ to their wives’ use of contraceptives compared to those with fewer or more children (Table 2). Similarly, the participants with one son were more supportive of contraceptive use compared to those with no son or with two or more sons. The participants of Hindu religion, belongs to other backward classes, living in well-off households, completed at least a higher secondary education were more supportive to their wives’ contraceptive use. Male involvement in supporting contraceptive use was higher among husbands with ‘pro-FP’, ‘pro-contraceptive’ and ‘supportive’ attitudes than their counterparts. Higher proportions of husbands who had higher exposure to FP messages through FLWs, or media were involved in decision making on family size.

### 3.4 Multivariable logistic regression analysis for predicting male involvement

Two separate multivariable logistic regression models (Table 3) were computed to identify the factors associated with husbands’ involvement in decision-making on family size with their wives, and their support for wives’ contraceptive use. Husbands with one (AOR = 1.96, 95% CI 1.23–3.13) or two (AOR = 1.90, 95% CI 1.14–3.18) living children were nearly twice as likely to be involved in decision-making on family size with their wives compared to those with no child. Participants with one son had 50% higher odds (AOR = 1.50, 95% CI 1.06–2.11) of discussing family size compared to those with two or more sons. Non-Hindu participants were 42% less likely to be involved in decision-making on family size (AOR = 0.58, 95% CI: 0.39–0.88) compared to participants who were Hindus. Husbands’ involvement in decision-making on family size increases with ‘pro-FP’ (AOR = 1.68, 95% CI 1.29–2.20) and ‘pro- contraceptive’ (AOR = 1.74, 95% CI 1.34–2.27) attitudes. Husbands who had got exposure from media regarding FP had 2.02 times odds (AOR = 2.02, 95% CI 1.53–2.66) of engaging themselves in decision-making on family size with their wives.

**Table 3:** Multiple logistic regression for predicting male involvement by the demographic, socio-economic, individualistic characteristics.

	Male involvement in decision on family size with wife	Male involvement to support contraceptive use with wife
	N = 1,258	N = 1,115
	AOR (95% CI)	AOR (95% CI)
**Explanatory variables**		
**Demographic characteristics**		
***Number of living Children***		
0	Ref.	Ref.
1	1.96** (1.23, 3.13)	1.33 (0.80, 2.20)
2	1.90* (1.14, 3.18)	1.74* (1.01, 3.01)
3+	1.37 (0.80, 2.34)	1.67 (0.94, 2.96)
**Number of sons**		
No son	1.32 (0.82, 2.10)	0.83 (0.52, 1.32)
One Son	1.50* (1.06, 2.11)	1.04 (0.74, 1.46)
Two or more than two sons	Ref.	Ref.
**Socio-economic characteristics**		
***Religion***		
Hindu	Ref.	Ref.
Non-Hindu	0.58* (0.39, 0.88)	0.57* (0.36, 0.88)
***Social-group***		
SC/ST	0.93 (0.65, 1.33)	0.88 (0.61, 1.27)
OBC	1.26 (0.91, 1.74)	1.33 (0.95, 1.85)
General	Ref.	Ref.
***Wealth index***		
Poor	0.87 (0.64, 1.19)	1.21 (0.88, 1.66)
Middle	Ref.	Ref.
Rich	1.31 (0.94, 1.83)	1.31 (0.93, 1.84)
***Education status***		
No education	Ref.	Ref.
Primary	1.02 (0.70, 1.48)	1.38 (0.94, 2.02)
Secondary	1.18 (0.84, 1.66)	1.34 (0.94, 1.91)
Higher	1.13 (0.73, 1.73)	1.40 (0.90, 2.19)
**Attitudinal characteristics**		
***Attitude toward family planning***		
Anti-FP	Ref.	Ref.
Pro-FP	1.68** (1.29, 2.20)	1.55** (1.17, 2.06)
***Contraceptive Attitude***		
Anti-contraceptive	Ref.	Ref.
Pro-contraceptive	1.74** (1.34, 2.27)	1.32* (1.01, 1.72)
***Attitude to support wife in contraceptive use***		
Non-supportive	Ref.	Ref.
Supportive	0.92 (0.71, 1.20)	2.05** (1.57, 2.68)
**Exposure to family planning messages**		
***Exposure from frontline health worker***		
No Exposure	Ref.	Ref.
Got Exposure	1.43 (0.86, 2.37)	1.59 (0.95, 2.66)
***Exposure from media***		
No Exposure	Ref.	Ref.
Got Exposure	2.02** (1.53, 2.66)	1.62** (1.23, 2.14)

* The AORs are significant at p < 0.05.

** The AORs are significant at p < 0.01.

Husbands with two living children were 74% more likely (AOR = 1.74, 95% CI 1.01–3.01) to support their wives in using contraceptives compared to those with no children. Non-Hindu husbands were 43% less likely (AOR = 0.57, 95% CI 0.36–0.88) to support their wives’ contraceptive use compared to Hindu husbands. Husbands with ‘pro-FP’ (AOR = 1.55, 95% CI 1.17–2.06), ‘pro-contraceptive use’ (AOR = 1.32, 95% CI 1.01–1.72), and ‘supportive’ (AOR = 2.05, 95% CI 1.57–2.68) attitude had significantly higher odds for involvement to support contraceptive use of their wife. Husbands who were exposed to FP messages through media were 62% more likely (AOR = 1.62, 95% CI 1.23–2.14) to support contraceptive use of their wives compared to those with no such exposure.

***Combined attitudinal variable:*** The regression analyses showed positive associations between all three attitudinal variables and both dependent variables. Therefore, a new combined attitudinal variable was created by conducting LCA using all three existing attitudinal variables. This variable was created for the ease of further analyses and to make the analyses more understandable for the program managers. Similar to other attitudinal characteristics, LCA model did not converge beyond two classes. The fit statistics for the model have been presented in S3 Appendix. The 50^th^ percentile of the predictive probabilities coincided with the marginal score of 0.6, therefore, used as the cut-off to classify the participants into two classes.

**Fig 3:**
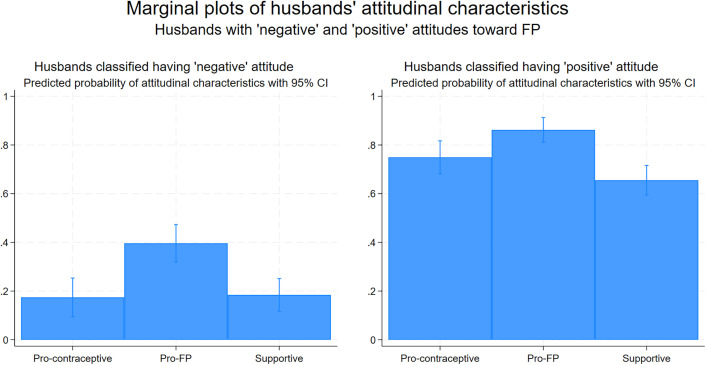
Marginal plots showing the predictive probability of attitudinal characteristics with 95% CI between two classes of husbands with ‘negative’ and ‘positive’ attitudes towards FP. Pro-contraceptive = positive attitude toward contraceptives, Pro-FP = positive attitude towards family planning, Supportive = positive attitude toward supporting contraceptive use of wives.

The postestimation marginal plots of the predictive probabilities of combined attitudinal variable have been presented in Fig 3, which shows the significant difference in predicted probabilities of favorable attitudinal characteristics between husbands of two latent classes. Husbands with lower predictive probabilities of favorable attitudinal characteristics are considered having ‘negative’ attitude toward FP and those with higher predictive probabilities are considered having ‘positive’ attitude, and this combined attitudinal variable has been used in further analyses.

This newly formed attitudinal variable classified the study participants into two groups, having ‘negative’ and ‘positive’ attitudes towards equal involvement in FP with their wives. This analysis classified 1,258 husbands into two groups: ‘negative’ (45%) and ‘positive’ (55%) based on their overall attitudes on FP.

We wanted to examine the characteristics of those husbands who had overall ‘positive’ attitudes. The distribution of socio-economic characteristics across husband’s overall attitude showed that the husbands with ‘positive’ attitudes were significantly more educated and belonged to either middle or rich wealth groups (Table 4). The analyses revealed a strong and significant association between husbands’ ‘positive’ attitude with the exposure to FP messages in the media (Chi square 30.18, p < 0.01). While 49% of husbands with no exposure of FP through media had ‘positive’ attitude, this increased to 64% among those exposed to any such messages, an increase of 15% points. However, we did not find any association of husband’s attitude with exposure to FP messages through FLWs.

**Table 4:** Background characteristics of husband with combined attitudinal variable.

	Husband having negative attitude (%)	Husband having positive attitude (%)	p-value
**Total Percentage**	**44.9**	**55.1**	
**Variables**			
***Age group***			0.544
18-24	47.6	52.5	
25-34	43.4	56.6	
More than 34	45.6	54.4	
***Educational Status***			0.011
No Education	51.4	48.6	
Primary	46.8	53.2	
Secondary	41.5	58.5	
Higher	39.8	60.2	
***Religion***			0.086
Hindu	44.1	55.9	
Non-Hindu	51.9	48.2	
***Social group***			0.743
SC/ST	45.3	54.7	
OBC	45.7	54.3	
General	43.0	56.9	
***Wealth Index***			< 0.001
Poor	52.8	47.2	
Middle	41.8	58.2	
Rich	40.3	59.7	
***Exposure from frontline health worker***			0.412
No Exposure	45.3	54.8	
Got Exposure	41.0	59.0	
***Exposure from media***			< 0.001
No Exposure	51.4	48.6	
Got Exposure	35.8	64.2	

The results of final models show that husbands’ ‘positive’ attitudes and exposure to FP messages are more likely to be involved in FP. This held true even after controlling significant determinants like the number of children or number of sons they had and the religious affiliation of husbands (Table 5). Even husbands with ‘negative’ attitudes towards FP showed significantly higher odds of engaging in decision-making on family size if they were exposed to FP messages through the media (AOR = 2.37, 95% CI 1.63–3.46), compared to those with similar attitudes but had no such exposure. ‘Positive’ attitudes alone increased odds of husbands’ involvement in decision making on family size by 86% (AOR = 1.86, 95% CI 1.38–2.52) compared to ‘negative’ attitudes with no media exposure, combining ‘positive’ attitudes with media exposure further increased the odds (AOR = 4.18, 95% CI 2.96–5.88).

**Table 5:** Multiple logistic regression for predicting male involvement by the demographic, socio-economic, individualistic characteristics.

	Husbands’ involvement in decision on family size with wife	Husbands’ involvement to support contraceptive use of wife
	N = 1,258	N = 1,115
	AOR (95% CI)	AOR (95% CI)
**Explanatory variables**		
**Demographic characteristics**		
***Number of living Children***		
0	Ref.	Ref.
1	1.93** (1.22, 3.06)	1.44 (0.88, 2.36)
2	1.71* (1.04, 2.83)	1.81* (1.07, 3.08)
3+	1.16 (0.69, 1.95)	1.65 (0.95, 2.85)
**Number of sons**		
No son	1.31 (0.83, 2.07)	0.85 (0.54, 1.33)
One Son	1.51* (1.08, 2.10)	1.06 (0.76, 1.48)
Two or more than two sons	Ref.	Ref.
**Socio-economic characteristics**		
***Religion***		
Hindu	Ref.	Ref.
Non-Hindu	0.57** (0.39, 0.84)	0.55* (0.36, 0.82)
***Interaction between attitude and exposure to FP through media***		
Negative & No Exposure	Ref.	Ref.
Negative & Got Exposure	2.37** (1.63, 3.46)	2.06** (1.40, 3.04)
Positive & No Exposure	1.86** (1.38, 2.52)	2.31** (1.69, 3.17)
Positive & Got Exposure	4.18** (2.96, 5.88)	3.98** (2.82, 5.61)

* The AORs are significant at p < 0.05.

** The AORs are significant at p < 0.01.

Similarly compared to husbands who had ‘negative’ attitude with no exposure to FP messages through the media, the odds for husbands’ involvement to support their wives’ contraceptive use were high for husbands with ‘negative’ attitude but media exposure (AOR = 2.06, 95% CI 1.40–3.04), husbands with ‘positive’ attitude but no media exposure (AOR = 1.69, 95% CI 1.69–3.17), and husband with both ‘positive’ attitude and media exposure (AOR = 3.98, 95% CI 2.82–5.61).

**Fig 4:**
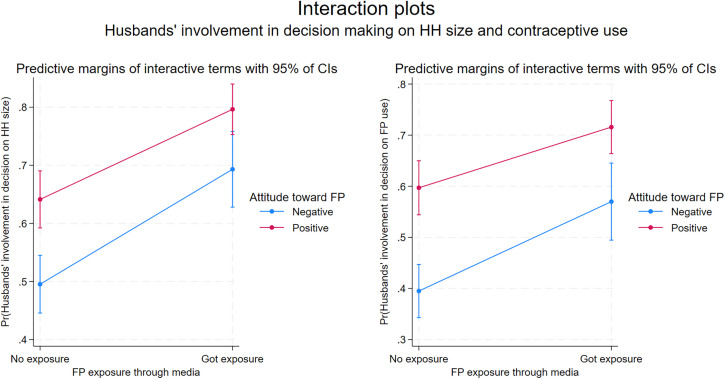
Post-estimation marginal plots showing the interaction effects of husbands’ combined attitude toward FP and exposure to FP messages through media on their involvement in FP.

The postestimation marginal plots of logistic regressions (Fig 4) graphically present the combined effect of attitude and media exposure, along with 95% of CIs, separately for two dependent variables of husbands’ involvement. The husbands with ‘positive’ attitude showed higher predictive probability of being involved in decision making on household size with their wives and a significantly higher predictive probability of being involved in supporting contraceptive use of their wives. Moreover, the husbands who were exposed to FP messages through media had higher predictive probability of getting involved. The interaction terms also showed the difference between husbands with ‘positive’ and ‘negative’ attitudes reduced if they are exposed to FP messages compared to those who were not exposed.

## 4. Discussion

This study assessed the level of husbands’ involvement in FP as partners to their wives. We also examined the factors associated with such involvement. The study area, two districts of Uttar Pradesh, historically known for high fertility, low use of modern contraceptives, and high unmet need [11,42,48]. Previous research has attributed poor FP outcomes to low male involvement. While earlier studies evaluated male involvement in FP, considering men as clients, this study evaluated husbands’ involvement as partners in contraceptive decision making and to support their wives in contraceptive use.

We found that nearly 65% of husbands were involved in decision-making on family size with their wives and 56% of husbands supported wives’ use of contraceptives. About two-thirds (66%) of husbands were ‘pro-FP’, with about half (50%) had a favorable attitude toward contraceptive use, and 47% had attitude to support their wives’ use of contraceptives. Only 8% received messages related to FP from health workers, and about 42% were exposed to FP through media.

Involvement in FP as higher among men having 1 or 2 children, or 1 son and this finding corroborates to the findings of earlier studies where the higher percentage of male involvement in FP has been reported among those who have at least one child and son [49,50]. Also, the male involvement in FP was higher among Hindus than non-Hindus. An earlier study in Bangladesh also reported similar association with religious background of the study participants [50]. In contrast to findings of Ijadunola et al. [51], Kamal et al. [19], and Rahayu et al. [52], who reported more positive attitude toward FP if husbands were more educated, non-Muslim, skilled or professional occupation, and belonged to higher wealth quintile, in our study no such association was found between husbands’ attitude with their socio-economic characteristics [19,51–53]. One of the reasons could be the inclusion of attitudinal characteristics in the analysis, unlike the previous studies.

Although the socio-economic characteristics like higher education, advantageous social groups, and greater wealth are generally linked to positive FP attitudes, inclusion of attitudinal factors in our analysis nullified the effect of socio-economic characteristics on husbands’ involvement in FP. This finding signifies that even the husbands with lower education, from disadvantageous groups, and less wealth could also be involved in FP with their wives. The interaction with the health workers on FP did not show any association to the husbands’ involvement, probably due to the very low percentage of husbands interacting with health workers on FP in the study area [20].

Consistent with the findings from previous research, the favorable attitudes of the husbands toward FP, use of contraceptives, and supporting wives’ contraceptive use showed most significant association with husbands’ involvement in FP. Although the literature on husbands’ attitudinal characteristics on their involvement in FP using LCA is limited, the previous research from Bangladesh, Ethiopia, India, Indonesia, Kenya, Naigeria, and Niger found higher use of contraceptives among class memberships with empowered, gender-egalitarian, and positive ideation factors [54–58].

Exposure to FP messages through media was another significant factor, associated with husbands’ involvement. Similar to studies among Indian women where media exposure to FP messages increased contraceptive use, especially the use of reversible modern methods, [39] this study too found a strong association between media exposure and husbands’ involvement in contraceptive use. These findings highlight the importance of leveraging media effectively for FP messaging in India. As media exposure demonstrably influences both women’s contraceptive use and men’s involvement, FP program managers and stakeholders should prioritize strategic media campaigns to reach a wider audience.

The husbands with a positive attitude toward FP tend to be more educated and come from well-off families. These findings corroborate with earlier studies where men’s positive attitude toward FP is associated to higher education, wealthier households, and having employment [23,59,60]. Our finding did not show association between husbands’ attitude and background characteristics like age, or social groups. This highlights that negative attitude can exist among husbands of from non-marginalized groups and positive attitudes can be found among men from disadvantaged and marginalized groups. Exposure to FP messages through media could be the reason for shaping the husbands’ attitude favorable toward FP across age and social groups. These are useful findings for the program implementation, and the program can leverage these “highly engaged husbands”—husbands from marginalized communities with positive attitudes—to promote the acceptance of FP within these communities.

**Fig 5:**
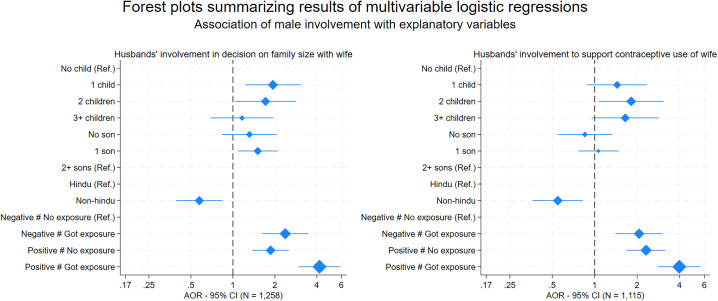
Forest plots explaining the association of male involvements in family planning with explanatory variables.

The results of final model have been visually presented in a set of forest plot diagrams (Fig 5). The husbands having ‘positive’ attitude toward FP with exposure to FP messages through media have the highest odds to get involved in FP even after controlling for number of children, having a son, and their religious background. The husbands with one child or without a son get involved in decision making for household size with their wives but their involvement to support wives’ contraceptive use is not significant.

The joint effect of positive attitude and media exposure had the most significant impact in husbands’ involvement in FP. This finding holds great promise for improving implementation of the FP programs. The higher exposure of FP messages in media had the potential to change husbands’ attitude toward more positive direction, ultimately leading to increased husbands’ involvement in FP.

### Limitation, strengths, and scope for future research

It is important to acknowledge that the categorization of husbands’ attitudes is based on a specific scoring system and may not fully capture nuances of individual perspectives or cultural variations. A particular strength of our study lies in focusing on region of the study—two districts, Fatehpur and Aligarh—from Uttar Pradesh, India’s most populous state. This regional focus provided valuable insights into the mechanics of husbands’ involvement in FP in the country.

Although this is a cross-section study where it is difficult to infer any changes over time, this study will serve as a baseline, allowing policymakers to assess the effects of existing policies on FP for future program planning. Another strength of this study is our extended definition of husbands’ involvement in FP. Unlike previous studies which were mostly confined to men’s contraceptive use we studied husbands’ involvement in a broader perspective, considering their involvement regarding joint-decision makings with their wives on FP and supporting wives’ contraceptive use.

We recoded the responses to contraceptive attitude scales into binary variables and this exercise is a deviance from the original scale’s scoring protocol. However, recoding was a crucial step for conducting multivariable logistic regression models. Further, the recoding of the data allowed to create interaction terms and improved overall model fit and predictive power by preventing statistical instability and allowing for more meaningful analysis [61].

The male involvement may get affected by some cofounding variables like spousal communication frequency on FP, age difference between husband and wife, or the nature of previous experience of contraceptive use, and existing norms in the society on contraceptive use. More detailed study in future including these variables could illuminate further understanding of male involvement in family planning.

Males who are already considering or are actively involved in family planning (e.g., using a method, attending FP discussions with a partner) may be more likely to exhibit selective attention. They may actively seek out, pay closer attention to, or better recall FP messages on television, radio, or social media compared to unengaged men. This suggests that prior involvement or even an intention to be involved could precede and inflate the observed level of media exposure, rather than media exposure being the sole driver of involvement.

The article hypothesizes that having a positive attitude and media exposure to messages on FP influences male involvement in FP. However, it is crucial to acknowledge the potential for reverse causation, where male involvement itself may lead to greater media exposure or their involvement can improve their attitude toward FP. Males who are already involved in FP may be more likely to exhibit selective attention. They may actively seek out, pay closer attention to, or better recall messages on television, radio, or social media compared to men who are less involved. Once a male gets involved, he might actively engage in information-seeking behavior to understand the methods better, learn about benefits, or address misconceptions. This targeted search for FP content (which is a form of media consumption) could be incorrectly interpreted as general media exposure driving the initial involvement.

Similarly, once a man gets involved in FP, he may subconsciously adjust his attitude to align with his behavior. This is a psychological mechanism often related to reducing cognitive dissonance, where a positive attitude is formed after the action to justify the decision and maintain consistency. Direct involvement leads to firsthand positive experiences, such as improved financial stability, better maternal/child health outcomes, or increased spousal communication. These tangible, positive results can fundamentally change a man’s perception, leading to a much stronger and more durable positive attitude toward FP. In this scenario, the action of involvement is the catalyst for the attitudinal shift. Given the potential for both types of reverse causation, establishing a definitive causal link based solely on cross-sectional data is methodologically challenging. Future research, particularly using longitudinal study designs (e.g., follow-up surveys, panel studies) or experimental/quasi-experimental approaches (e.g., randomized control trials of media campaigns), would be necessary to establish the temporal ordering of events and more robustly test the direction of causality.

The key strength of this study is our investigation into the role of husbands’ attitudinal characteristics on their involvement in FP, however, because of self-reported data the study may be affected by social desirability bias, where respondents may indicate more positive behaviors or attitudes than they exhibit. Our findings highlight the importance of men’s attitude towards FP and contraceptive use, providing insights that go beyond socio-economic factors which will pave course of future research for programmatic importance.

The scope of this study corroborates to the recent emphasis on FP programming by the Government of India engaging male [1]. For policymakers and program implementers, the study’s emphasis on attitudinal characteristics and involvement of male partners in FP decision-making offer insightful information. The study indicates that focused media efforts might considerably increase male involvement in FP by highlighting the combined influence of favorable attitudes and media exposure. These results notably apply to areas with comparable socio-cultural and demographic backgrounds, providing a guide for enhancing male involvement in FP and, in turn, improving reproductive health outcomes.

Findings of our study highlight the need for FP programs to address underlying gender norms and masculinity constructs that shape men’s attitudes and behaviors. Men’s attitudes toward FP are deeply embedded within prevailing gender norms and masculinity ideals. In many contexts, hegemonic masculinity positions men as primary decision-makers and associates virility with having multiple children, which can create resistance to contraceptive use. Conversely, positive attitudes toward FP often reflect a shift toward more gender-equitable masculinities, where men embrace shared responsibility for reproductive health and prioritize the well-being of their partners and families.

The existing program on FP could identify men with positive attitude toward FP and contraceptive use and leverage them efficiently in program implementation. Identifying men with a positive attitude in the community requires a structured approach with a mix of behavioral, and social indicators. Behavioral characteristics could include vising health facility with wife, participating in health meetings within community; while frontline health workers, women and community leaders, or peer groups could identify such men who has positive attitude and would be ready to be engaged in FP programming. Interventions should move beyond providing contraceptive information to actively challenge norms that equate masculinity with dominance, fertility control, and virility. Programs can leverage positive masculinity by promoting narratives of responsible fatherhood, shared decision-making, and care for partners. Strategies such as engaging male champions, incorporating gender-transformative messaging, real-life stories and testimonials, and creating safe spaces for men to discuss reproductive health can help normalize supportive attitudes and foster equitable participation in FP.

## 5. Conclusions

The importance of husbands’ involvement in FP programs is well-recognized, and program managers endorse this approach. However, historical trends and entrenched gender norms in Uttar Pradesh have made policymakers and program managers skeptical about effective involvement strategies. This study offers encouraging findings as a significant proportion of husbands jointly participate in FP decisions with their wives and support contraceptive use, demonstrating positive attitudes. Furthermore, the “highly engaged husbands”-husbands from disadvantaged communities with positive attitudes towards FP-offer a valuable opportunity. By leveraging these individuals, programs can address social barriers against contraceptive use within these communities. This approach could lead to a reducing unmet need among married women, improving access to contraceptives, and increasing contraceptive continuation rates, ultimately leading to better maternal health outcomes.

## Supporting information

S1. Appendix Description of the study and explanatory variables. (PDF)

S2. Appendix Fit indices of latent class analyses for husbands’ attitude to support wife in contraceptive use, attitude toward family planning, and combined attitude variable. (PDF)

S3. Appendix Marginal plots showing the predictive probability of attitudinal characteristics with 95% CI between two classes of husbands with ‘negative’ and ‘positive’ attitudes towards FP. Note: [given in table].
(PDF)

S1 File Response variables. (DOCX)
