# Supplementary material for: Determinants of husbands’ involvement in family planning: Evidence from a community-based cross-sectional study in Uttar Pradesh, India
Source: PLoS One. 2026 Apr 29;21(4):e0343591. doi: 10.1371/journal.pone.0343591 (PMC13127951; doi:10.1371/journal.pone.0343591)
Supplement: S1. Appendix — (PDF) [file pone.0343591.s001.pdf]

Appendix 1. Description of the study and explanatory variables

Variable	*Response*	*Recoding*
Outcome variables		
Male involvement in decision on family size		
Have you ever discussed about number of children to have with your wife?	1 Yes, 2 No	
Have you ever discussed about spacing children with your wife?	1 Yes, 2 No	
Male involvement to support contraceptive use		
Have you ever discussed contraceptive methods with your wife?	1 Yes, 2 No	
Would you say that using contraception was mainly your decision, mainly your wife's decision, or you both decided together or someone else took the decision?<br>*Or*<br>*Would you say that not using any contraception is mainly your decision, mainly your wife's decision, or you both decided together or someone else took the decision?*	Mainly Husband=1<br>Mainly wife = 2<br>Jointly = 3<br>Someone else=4<br>OR<br>Mainly Husband=1<br>Mainly wife = 2<br>Jointly = 3<br>Someone else=4	2/3 in 407 or 2/3 in 418-> 1<br><br>Otherwise->0
Explanatory variables		
Demographic characteristics		
Number of living children	Number of sons= 0,1,2,3,4,5,6.<br>Number of daughters= 0,1,2,3,4,5,6,7.	0 No child<br>1 One child<br>2 Two children<br>3 or 3+ children
Number of sons	Number of sons= 0,1,2,3,4,5,6.	0 'No son'<br>At least one son
Socio-economic characteristics		
Religion	1 'Hindu', 2 'Muslim', 3 'Other'	1 Hindu<br>2 non-Hindu
Caste	1 'SC', 2 'ST', 3'OBC', 4 'None of them'	1 SC/St<br>2 OBC<br>3 General/ Other
Wealth index	1 Poor, 2 Middle, 3 Rich	
Education	Highest level of Education- '0 to 17' years.	0 'No education'.<br>'1-5' Primary.<br>'6-10' Secondary. 10+ Higher.
Attitudinal characteristics		
Attitude towards family planning		
According to you, how important is it for couples to avoid an unwanted pregnancy. What would you say, important, somewhat important, or not at all important?	1 Important<br>2 somewhat important<br>3 not at all important	1->1 Important.<br>Otherwise, 0 Not important.
Should couple start using a FP method immediately after marriage/starting to stay together/ before first child?	1 Yes, 2 No	
Should couple start using a FP method after birth of first child?	1 Yes, 2 No	
Should couple use a FP method after completing their desired family sizes?	1 Yes, 2 No	

Should a couple use a FP method if they have only daughters?	1 Yes, 2 No	
Contraceptive attitude scale		
Contraception should only be women's responsibility<br>*Unmarried girls should not be provided knowledge on contraceptives*<br>*Unmarried girls should not be given contraceptives*<br>*Providers should decide which contraceptive is suitable for a woman*<br>*Use of condom is a sign of infidelity between husband and wife*<br>*Husband should have final say on contraceptive use*<br>*Women should seek approval from their in-laws to initiate a contraceptive*	1 'Strongly Agree'<br>2 'Agree'<br>3 'Disagree'<br>4 'Strongly Disagree'	'Strongly Agree,' 'Agree' ->0<br><br>Otherwise ->1
Men should actively take responsibilities for contraception<br>*Men should acquire as much knowledge on FP as they can*<br>*Women should acquire as much knowledge on FP as they can*<br>*Women should have the right to decide when and which method to use*<br>*Privacy of all clients seeking FP services, irrespective of their age, sex, caste and religion, should be protected*	1 'Strongly Agree'<br>2 'Agree'<br>3 'Disagree'<br>4 'Strongly Disagree'	'Strongly Agree,' 'Agree' ->1<br><br>Otherwise ->0
Attitude to support wife in contraceptive use		
Suppose you want to use an FP method and your wife doesn't agree, who would make the final decision?	1 'Myself'<br>2 'Wife'	Myself->0<br>Wife->1
Suppose your wife wants to use an FP method and you do not agree, who would make the final decision?	1 'Myself'<br>2 'Wife'	Myself->0<br>Wife->1
If your wife wanted to use a family planning method in order to plan (space) births, would you agree with her?	1 'Yes'<br>2 'No'	Yes ->1<br>Otherwise ->0
Do you feel confident that you and your wife could use a FP method, even if other people in your community ridicule you for using FP method? Would you strongly agree, agree, disagree or strongly disagree?	1 'Strongly Agree'<br>2 'Agree'<br>3 'Disagree'<br>4 'Strongly Disagree'	'Strongly Agree', 'Agree' ->1<br>Otherwise ->0
Do you think that women who use contraceptive is promiscuous?	1 'Strongly Agree'<br>2 'Agree'<br>3 'Disagree'<br>4 'Strongly Disagree'	Strongly Agree', 'Agree' ->0<br>Otherwise ->1
As you said, avoiding unwanted pregnancy is important and sexual pleasure is also important in a husband-wife relationship, so If a couple had to choose an FP method that can affect sexual pleasure but prevents unwanted pregnancy, should they still accept that FP method?	1'Yes'<br>2' No'	Yes ->1<br>Otherwise ->0
Exposure of FP messages through frontline health worker		
In last 12 months, have you met with ASHA/AWW/ANM?<br>*and*<br>*During (this contact/all these contacts) with (PERSONS MENTIONED) in the last 12 months, what were discussed, or services received?*	A-C Different cadre of FLW, D-None<br><br>A – Family planning	(A, B, or C) in 601 and A in 602 or 1 in 609 -> 1<br>Otherwise ->0
Did you receive any information or service regarding family planning at the health	1'Respondent'<br>2 'Husband'	

facility in last 12 months?		
Exposure of FP messages through media		
Do you read newspaper(s)/magazines? *and* *Have you read any family planning related information in newspapers/magazines in the past three months?*	1 Yes, 2 No<br>1 Yes, 2 No	1 = 701 and 1 = 702 -> 1<br>Otherwise, 0
Do you listen to the radio? If yes, how often would you say almost every day, at least once a week, at least once a month, rarely or not at all *and* *Have you heard any family planning information on the radio in the past three months?*	1-4 'Frequencies of radio listening'<br>8 'Not at all'<br>1 Yes, 2 No	1-4 in 703 and 1 in 705 -> 1<br>Otherwise, 0
Do you watch television? *And* *Have you seen any family planning information on the TV in the past three months?*	1 Yes, 2 No<br>1 Yes, 2 No	1 in 706 and 1 in 709-> 1<br>Otherwise- 0
Have you seen any family planning information on a wall painting or hoarding in the past three months?	1 Yes, 2 No	
Do you use internet? *And* *Have you seen any family planning information on the internet in the past three months?*	1 Yes, 2 No<br>1 Yes, 2 No	1 in 711 and 1 in 713-> 1<br>Otherwise- 0
