# Supplementary material for: Determinants of husbands’ involvement in family planning: Evidence from a community-based cross-sectional study in Uttar Pradesh, India
Source: PLoS One. 2026 Apr 29;21(4):e0343591. doi: 10.1371/journal.pone.0343591 (PMC13127951; doi:10.1371/journal.pone.0343591)
Supplement: S2. Appendix — (PDF) [file pone.0343591.s002.pdf]

Appendix 2. Fit indices of latent class analyses for husbands' attitude to support wife in contraceptive use, attitude toward family planning, and combined attitude variable

	**Log-likelihood**	**df**	**AIC**	**BIC**	**Entropy**
**Attitude to support wife in contraceptive use**					
One class	-2845.632	5	5701.264	5726.951	-
Two class	-2433.859	11	4889.719	4946.229	0.909
**Attitude toward family planning**					
One class	-4585.374	8	9186.748	9227.846	-
Two class	-4416.834	17	8867.667	8955.001	0.712
**Combined attitudinal variable**					
One class	-2543.496	3	5092.993	5108.405	-
Two class	-2430.703	7	4875.405	4911.366	0.479
