# Supplementary material for: Determinants of husbands’ involvement in family planning: Evidence from a community-based cross-sectional study in Uttar Pradesh, India
Source: PLoS One. 2026 Apr 29;21(4):e0343591. doi: 10.1371/journal.pone.0343591 (PMC13127951; doi:10.1371/journal.pone.0343591)
Supplement: S3. Appendix — Note: [given in table]. (PDF) [file pone.0343591.s003.pdf]

Appendix 3. Marginal plots showing the predictive probability of attitudinal characteristics with 95% CI between two classes of husbands with 'negative' and 'positive' attitudes towards FP

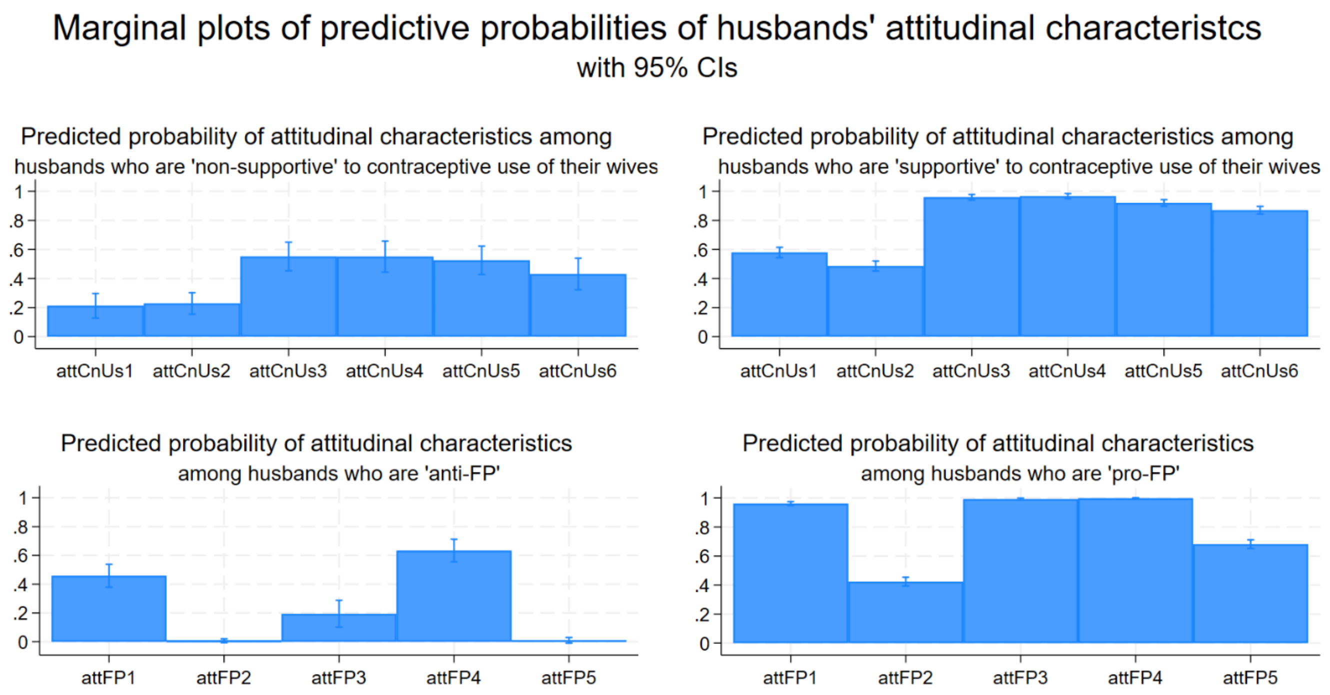

Note:

**Response variable**	**Presented in figure**
Attitudinal characteristics of husbands to support wife in contraceptive use	
Suppose you want to use an FP method and your wife doesn't agree, who would make the final decision? Ans. wife	attCnUs1
Suppose your wife wants to use an FP method and you do not agree, who would make the final decision? Ans. wife	attCnUs2
If your wife wanted to use a family planning method in order to plan (space) births, would you agree with her? Ans. Yes	attCnUs3
Do you feel confident that you and your wife could use a FP method, even if other people in your community ridicule you for using FP method? Would you strongly agree, agree, disagree or strongly disagree? Ans. disagree or strongly disagree	attCnUs4
Do you think that women who use contraceptive is promiscuous? Ans. disagree or strongly disagree	attCnUs5
As you said, avoiding unwanted pregnancy is important and sexual pleasure is also important in a husband-wife relationship, so if a couple had to choose an FP method that can affect sexual pleasure but prevents unwanted pregnancy, should they still accept that FP method? Ans. Yes	attCnUs6
Attitude toward family planning	
According to you, how important is it for couples to avoid an unwanted pregnancy. What would you say, important, somewhat important, or not at all important? Ans. Important	attFP1
Should couple start using a FP method immediately after marriage/starting to stay together/ before first child? Ans. Yes	attFP2
Should couple start using a FP method after birth of first child? Ans. Yes	attFP3
Should couple use a FP method after completing their desired family sizes? Ans. Yes	attFP4
Should a couple use a FP method if they have only daughters? Ans. Yes	attFP5
