# Supplementary material for: Determinants of husbands’ involvement in family planning: Evidence from a community-based cross-sectional study in Uttar Pradesh, India
Source: PLoS One. 2026 Apr 29;21(4):e0343591. doi: 10.1371/journal.pone.0343591 (PMC13127951; doi:10.1371/journal.pone.0343591)
Supplement: S1 File — (DOCX) [file pone.0343591.s004.docx]

| **Response variable** | **Presented in figure** |
| --- | --- |
| ***Attitudinal characteristics of husbands to support wife in contraceptive use*** |  |
| Suppose you want to use an FP method and your wife doesn’t agree, who would make the final decision? Ans. wife | attCnUs1 |
| Suppose your wife wants to use an FP method and you do not agree, who would make the final decision? Ans. wife | attCnUs2 |
| If your wife wanted to use a family planning method in order to plan (space) births, would you agree with her? Ans. Yes | attCnUs3 |
| Do you feel confident that you and your wife could use a FP method, even if other people in your community ridicule you for using FP method? Would you strongly agree, agree, disagree or strongly disagree? Ans. disagree or strongly disagree | attCnUs4 |
| Do you think that women who use contraceptive is promiscuous? Ans. disagree or strongly disagree | attCnUs5 |
| As you said, avoiding unwanted pregnancy is important and sexual pleasure is also important in a husband-wife relationship, so if a couple had to choose an FP method that can affect sexual pleasure but prevents unwanted pregnancy, should they still accept that FP method? Ans. Yes | attCnUs6 |
| ***Attitude toward family planning*** |  |
| According to you, how important is it for couples to avoid an unwanted pregnancy. What would you say, important, somewhat important, or not at all important? Ans. Important | attFP1 |
| Should couple start using a FP method immediately after marriage/starting to stay together/ before first child? Ans. Yes | attFP2 |
| Should couple start using a FP method after birth of first child? Ans. Yes | attFP3 |
| Should couple use a FP method after completing their desired family sizes? Ans. Yes | attFP4 |
| Should a couple use a FP method if they have only daughters? Ans. Yes | attFP5 |
